# Why caretakers bypass Primary Health Care facilities for child care - a case from rural Tanzania

**DOI:** 10.1186/1472-6963-11-315

**Published:** 2011-11-17

**Authors:** Catherine Kahabuka, Gunnar Kvåle, Karen Marie Moland, Sven Gudmund Hinderaker

**Affiliations:** 1 Centre for International Health, Faculty of Medicine and Dentistry, University of Bergen, Norway; 2 Department of Nursing, Bergen University College, Norway

## Abstract

**Background:**

Research on health care utilization in low income countries suggests that patients frequently bypass PHC facilities in favour of higher-level hospitals - despite substantial additional time and financial costs. There are limited number of studies focusing on user's experiences at such facilities and reasons for bypassing them. This study aimed to identify factors associated with bypassing PHC facilities among caretakers seeking care for their underfive children and to explore experiences at such facilities among those who utilize them.

**Methods:**

The study employed a mixed-method approach consisting of an interviewer administered questionnaires and in-depth interviews among selected care-takers seeking care for their underfive children at Korogwe and Muheza district hospitals in north-eastern Tanzania.

**Results:**

The questionnaire survey included 560 caretakers. Of these 30 in-depth interviews were conducted. Fifty nine percent (206/348) of caretakers had not utilized their nearer PHC facilities during the index child's sickness episode. The reasons given for bypassing PHC facilities were lack of possibilities for diagnostic facilities (42.2%), lack of drugs (15.5%), closed health facility (10.2%), poor services (9.7%) and lack of skilled health workers (3.4%). In a regression model, the frequency of bypassing a PHC facility for child care increased significantly with decreasing travel time to the district hospital, shorter duration of symptoms and low disease severity.

Findings from the in-depth interviews revealed how the lack of quality services at PHC facilities caused delays in accessing appropriate care and how the experiences of inadequate care caused users to lose trust in them.

**Conclusion:**

The observation that people are willing to travel long distances to get better quality services calls for health policies that prioritize quality of care before quantity. In a situation with limited resources, utilizing available resources to improve quality of care at available facilities could be more appropriate for improving access to health care than increasing the number of facilities. This would also improve equity in health care access since the poor who can not afford travelling costs will then get access to quality services at their nearer PHC facilities.

## Background

Limited access to available health interventions, especially for children from poor families and communities, is among the main factors suggested to be associated with child deaths worldwide and especially in Africa [1]. Approximately 8.8 million children under the age of five die every year worldwide, the majority from infectious diseases (malaria, pneumonia and diarrhoea) largely amenable to currently available preventive and curative interventions [2]. A neonatal series report published in the Lancet in 2005 estimated that a reduction in neonatal deaths of up to 72% could be achieved through already available interventions, if provided at high coverage [3]. Furthermore, many studies have demonstrated a significant decrease in child mortality following general improvements in primary health care coverage. In Liberia and Zaire, sharp declines in child death (19% and 32%, respectively between 1984 and 1989) coincided with the introduction of intensive child health care programmes [4]. In a region of Senegal the child death rate fell from 350 to 81 per 1000 live births over a 25-year period in parallel with the implementation of modern health services [5]. Studies showing a large fraction of children dying at home also indicate under-utilization of health care services [6].

To facilitate access to health care, many low-income countries have assigned village-level PHC facilities as the main point of care for uncomplicated health problems. These are usually run by non-physician clinicians, such as clinical officers and/or nurses who are trained to attend simple cases and refer complicated ones to hospitals. However, research suggests that patients frequently bypass these facilities in favour of higher level hospitals despite substantial additional time and financial costs. A study in Kenya found that half or more of survey respondents bypassed their nearest (usually lower level) facility for antenatal care, immunization and child illnesses [7]. High bypass for outpatient care of episodic common illnesses has also been observed in Sri-Lanka and Namibia [8,9].

In recent years, Tanzania has significantly increased the number of health facilities across the country with the primary aim of improving access to health care for its entire people. The new Tanzanian health policy proposes establishment of a dispensary in every village, a health centre in every ward and a district hospital in every district. By 1992 about 72% of the population lived within 5 km of a health facility and 93% lived within 10 km [10]. However, the problem of bypass has also been reported in Tanzania [11,12].

The main reasons given for bypassing PHC facilities in the studies mentioned above were poor services (including lack of drugs and diagnostic services) and lack of trust in health workers at the bypassed facilities [7,11,12]. None of the studies explored in-depthly the actual user's experiences at such facilities in relation to the reported deficiencies. Focusing on utilization of PHC facilities among care-takers seeking care for their underfive children in rural Tanzania, this study aimed to 1) establish the frequency of bypassing such facilities 2) identify factors influencing bypass and, 3) explore experiences, at such facilities, of caretakers seeking care for their under-five children.

## Methods

### Study area

The study was conducted at Korogwe and Muheza district hospitals of Tanga Region in north-eastern Tanzania. The two hospitals serve as referral hospitals in the two districts. Korogwe district is located about 100 km inland from Tanga. Based on the 2002 Census and a population growth rate of 1.2% per year [13], the projected population in 2009 was 282,901. The district is served by 47 dispensaries, four health centres and three hospitals (the study hospital and two church owned hospitals. Muheza district is around 25 km from the city of Tanga and about 70 km from Korogwe. Based on 2002 census and a population growth rate of 1.4%, it had a projected population of 306,862 in 2009 [13], which is served by 54 dispensaries, four health centres, and one district hospital. The two districts were purposely selected because they are endemic for malaria which is the leading cause of admissions and deaths among under-five children in both districts [6,14]. They are also predominantly rural, but were still easy to access.

### Study design

The study employed concurrent mixed method approach applying both quantitative and qualitative research methods. The quantitative part focused on establishing the frequency and reasons for bypassing PHC facilities. This was complemented by in-depth interviews which explored perceptions and experiences related to the quality of care offered at PHC facilities. The actual sample size for the quantitative part of this study was calculated for another purpose. Analysis of the 348 children included in this study gave a power of 80% to determine a PHC bypassing difference of 15% between two equally large groups of children (60% versus 45%), granted a 95% CI. Data were collected between July 2009 and January 2010.

#### Quantitative data collection

The target population were care-takers of sick children between 1 month and 5 years, with a diagnosis of malaria, pneumonia or diarrhoea seeking care at the outpatient departments (OPDs) of the two district hospitals. We identified all sick children presenting at the OPD, between 9 am and 2 pm, with a history of one or more of the following symptoms; fever, cough, difficulty in breathing/fast breathing, diarrhoea or vomiting. These were reviewed and assigned diagnosis as per WHO guidelines [15] by the principal investigator or a trained clinical officer. Children with a diagnosis of malaria, pneumonia or diarrhoea were included in the study. Some children with a provisional diagnosis of malaria were also included even if the subsequent result of malaria rapid test was negative. Caretakers of children who needed admission to the hospital were interviewed later in the ward after the children had received treatment. Caretakers of severely ill children who died before the interviews could be conducted were not included.

Interviews were conducted by trained clinical officers using a pre-tested questionnaire after obtaining a written informed consent. We obtained detailed care-seeking information pertaining to the current child's sickness episode including all treatments received and their sources, as well as availability and use of PHC facilities. When the caretaker had reported presence of a PHC facility nearer than the district (study) hospital but had not at all utilized it during the index child's sickness episode, an open-ended question was used to investigate reasons for bypassing it. Other information collected included questions on indicators of socio-economic status such as household characteristics, composition and assets.

#### In-depth interviews

In-depth interviews were conducted with the primary aim of exploring possible contributions of different potential factors (including quality of services offered at PHC facilities) to child's progression to severe disease. Hence, these were only conducted with caretakers who at the day of admission presented with a severely ill child. All children with a confirmed diagnosis of severe malaria, very severe pneumonia or acute watery diarrhoea with severe dehydration qualified to be interviewed. Caretakers were asked to recall details on actions taken from when they recognised the first symptom of the current child's sickness until the day of admission. Those who utilized their nearer PHC facilities were asked to give full details of what happened at these facilities while those who did not utilize them were asked to give reasons for bypassing them. A total of 30 in-depth interviews were conducted at around which point nothing new seemed to be coming out of the interviews. The interviews lasted between 25 and 45 minutes and were all performed by the principle investigator in Swahili, the national language. All interviews were digital-recorded and all the interviewees were also part of the quantitative survey.

### Ethical consideration

Ethical approval was obtained from the National Institute for Medical Research in Tanzania. Informed written consent was obtained from all study participants prior to conducting the interviews. No caretaker refused to participate, neither for the quantitative nor the qualitative interviews. All study procedures were conducted with caution not to interfere with the patient's ordinary consultations and only after the child had received all the necessary treatment.

### Data analysis

The quantitative data were double-entered and validated using Epidata version 3.1. SPSS version 18 was used for final analysis. A bypasser was defined as a caretakers who reported to have a PHC facility (dispensary or health centre) nearer than the district hospital but did not utilize it at all during the current child's sickness episode. When the district hospital was the nearest facility, the care-taker was classified as a non-bypasser.

We performed bivariate analysis to examine associations between bypassing a nearer facility and potential predictors, and multivariate analyses to determine which predictors remained associated with bypass when adjusted for other factors. The variables selected for the multivariate model were either significant (p-value < 0.05) in the bivariate analysis or shown to be significant in previously published studies [16-18].

The analysis of the qualitative data followed the principles of thematic content analysis. The interviews were transcribed and analysed in Swahili language. The data material was coded and categorised according to the recurrent themes. Translation into English was done at a later stage after identifying meaningful themes.

## Results

### Socio-demographic characteristics

Table 1 depicts some of the background characteristics of our study participants. A total of 560 caretakers were interviewed in the hospital survey, 52.5% from Muheza and 47.5% from Korogwe. The majority of respondents were biological mothers (92.1%), were married (78.6%) and peasants (59.8%). The overall mean age of respondents was 28.3 years, with the majority (85.5%) between 18 and 35 years. The majority (75.0%) had primary school education and 58.8% had completed it. Only 83 (14.8%) reported to have electricity and the majority (72.7%) used firewood as the main fuel for cooking. Approximately half of children were male (53.8%) and the majority (64.7%) were less than two years of age.

**Table 1:** Characteristics of interviewed child-caretakers in Korogwe and Muheza districts in 2009/2010 (n = 560)

Characteristic	Quantitative	Qualitative
	No. (%)	No. (%)
**Study site**		
Korogwe	294 (52.5)	17 (56.7)
Muheza	266 (47.5)	13 (43.3)
**Child sex**		
Male	301 (53.8)	14 (46.7)
Female	259 (46.3)	16 (53.3)
**Child age (in months)**		
0-11	210 (37.6)	3 (10.0)
12-23	151 (27.1)	7 (23.3)
24+	197 (35.3)	20 (66.7)
**Care-taker's level of education**		
No formal education	101 (18.0)	10 (33.3)
Primary education (std I-VII)	420 (75.0)	18 (60.0)
Post-primary education	39 (7.0)	2 (6.7)
**Care-taker's occupation**		
Housewife/Househusband	106 (18.9)	2 (6.7)
Peasant	335 (59.8)	25 (83.3)
Small scale business	96 (17.1)	1 (3.3)
Employed	17 (3.0)	2 (6.7)
Others	6 (1.2)	0 (0)
**Care-taker's relationship to the child**		
Biological mother	515 (92.1)	22 (73.3)
Biological father	17 (3.0)	4 (13.3)
Grandmother	15 (2.7)	2 (6.7)
Grandfather	1 (0.2)	0 (0)
Others	11 (2.0)	2 (6.7)
**Total**	**560 (100)**	**30 (100)**

We have full information everywhere except for 1 child under "care-taker's relationship to the child" and 2 children under "child age".

The background characteristics of the 30 caretakers participating in the qualitative study were very similar to the bigger study group except for the age distribution of the children, a majority (66.7%) being two years or more.

The majority of children whose caretakers underwent in-depth interviews had a diagnosis of severe malaria (17/30). Five had very severe pneumonia, five had acute watery diarrhoea (AWD) with severe dehydration, one had both severe malaria and AWD with severe dehydration while two had both very severe pneumonia and AWD with severe dehydration.

### Care seeking patterns

Almost all caretakers (91.6%) reported taking some action within the first 24 hrs after recognizing the first child's symptom. The majority (52.1%) gave simple treatment, mainly paracetamol, purchased from local or drug shops. A few (14.5%) gave some treatments available at home, commonly from previous consultations. Only 20.9% went to public hospitals within the first 24 hrs.

For the majority, the main reason given for the first action taken was that it was the closest available care (55.6%). However, a larger proportion of those who utilized public hospitals as the first option of care said it was the care they trusted more as compared to those who used other forms of care (36.8% versus 5.3%).

Treatment given at home originating from previous consultations included paracetamol, oral rehydration fluid, metronidazole, zinc, salbutamol, cotrimoxazole, quinine, co-artem and amoxicillin. Most of these drugs were also reported to be purchased from local shops.

### Bypass of PHC facilities

In the Quantitative survey, 348 caretakers (62.7% of the total material) reported having a nearer facility other than the study hospital. Of these, 206 (59.2%) had bypassed them during the current child's sickness episode. Among 30 caretakers interviewed qualitatively, 26 reported to have a PHC facility closer to home than the district (study) hospital. Similarly 12 had bypassed this facility.

Table 2 below depicts associations between potential factors associated with bypass among caretakers who reported a nearer PHC facility in the quantitative survey. The results showed no significant difference between the frequency of caretakers bypassing their nearest facility according to child's age and sex, and caretaker's socio-economic status (Table 2). We found significant associations between bypassing a PHC facility and study site (66.1% in Korogwe district compared to 52.5% in Muheza district) and caretaker's level of education (83.3% among caretakers with post-secondary education compared to 52.7% among those with no formal education). However, the association with education disappeared after controlling for other factors. We found persistent inverse associations between bypassing a PHC facility and travel time to the district hospital, child's duration of symptoms and child's disease severity (Table 2).

**Table 2:** Factors influencing the odds of bypassing a nearer facility by caretakers in the two districts (n = 348)

Risk factor for bypassing	Reported nearer facility	Bypassers	**OR**^**1**^	**AOR**^**2**^
	**n = 348**	**n = 206**	**(95% CI)**	**(95% CI)**
**Study Site**				
Korogwe	171	113 (66.1)	1.8 (1.1-2.7)*	1.8 (1.0-3.3)*
Muheza	177	93 (52.5)	Ref	Ref
**Child sex**				
Female	155	88 (56.8)	Ref	Ref
Male	193	118 (61.1)	1.2 (0.8-1.8)	1.5 (0.8-2.8)
**Child age (months)**				
0-11	127	73 (57.5)	Ref	Ref
12-23	88	55 (62.5)	1.2 (0.7-2.1)	1.4 (0.6-3.0)
24+	132	77 (58.3)	1.0 (0.6-1.7)	1.1 (0.5-2.3)
**Number of living children**				
1 child	86	59 (68.6)	2.0 (1.1-3.8)*	1.1 (0.5-2.7)
2-3 children	168	98 (58.3)	1.3 (0.8-2.2)	1.1 (0.5-2.4)
4 or more	91	47 (51.6)	Ref	Ref
**Caretakers Level of Education**				
No formal education	74	39 (52.7)	Ref	Ref
Primary education	256	152 (59.4)	1.3 (0.8-2.2)	1.0 (0.5-2.2)
Post-primary education.	18	15 (83.3)	4.5 (1.2-16.8)*	1.4 (0.3-6.9)
**SES**				
Low	108	59 (54.6)	Ref	Ref
Middle or high	240	147 (61.2)	1.3 (0.8-2.1)	1.5 (0.8-2.8)
**Travel time to district hospital**				
0-59 minutes	73	56 (77.0)	3.1 (1.6-5.8)*	3.5 (1.5-8.2)*
60-119 minutes	110	80 (72.7)	2.4 (1.4-4.2)*	2.4 (1.2-4.8)*
120+ minutes	126	66 (52.4)	Ref	Ref
**Symptoms duration**				
1-2 days	167	131 (78.4)	8.0 (4.4-14.4)*	11.0 (5.1-23.7)*
3-4 days	98	49 (50.0)	2.2 (1.2-4.0)*	5.0 (2.2-11.2)*
5 or more days	85	28 (31.3)	Ref	Ref
**Disease Severity**^3^				
Non-severe	120	98 (81.7)	5.3 (3.1-9.0)*	4.5 (2.2-9.1)*
Severe disease	203	93 (45.8)	Ref	Ref

* - Significant findings

1 Unadjusted Odds Ratio from bivariate analysis

2 Adjusted Odds Ratio for all other variables

3 Non-severe disease above includes: Non-severe malaria, non-severe pneumonia and acute watery diarrhoea with no dehydration

Severe disease included: Severe malaria, moderately severe and very severe pneumonia, and acute watery diarrhoea with some and with severe dehydration.

At the district hospital, majority of children (81.7%) seen with non-severe disease had bypassed their PHC facilities, as compared to less than half (45.8%) among those with severe disease.

### Reported quality of care at PHC facilities

Similar concerns related to availability and quality of care at PHC facilities were raised in the quantitative survey as well as the qualitative interviews. In the quantitative survey, lack of diagnostic facilities was mentioned most frequently by study participants as the main reason for bypassing the PHC facilities (42.2%). This was followed by lack of drugs (15.5%), the PHC facility being closed (10.2%) and services at the PHC facility not being good (9.7%). A few caretakers (3.4%) mentioned unavailability of staff as one of the reasons for bypassing PHC facilities while eighty participants (38.8%) gave other numerous reasons different from above, each comprising less than one percent.

The recurrent themes of the interviews also revealed lack of diagnostic facilities and drugs as being among the multiple deficiencies that care takers expected to encounter or had encountered at their nearer PHC facility. The following case is based on a mother's account of the care offered to her sick child at PHC facilities and illustrates how these 'multiple lacks' cause delays that may be fatal.

#### Case 1

A mother was seen at the district hospital 4 days after the onset of symptoms of her 4 months old female baby. On admission the child was semiconscious, dyspnoeic and had severe hypoglycaemia. The child died 8 hrs after admission. Below is a day to day account of the development of the sickness based on the mother's experiences of the care received at the PHC facilities where she first sought help for her sick baby.

**Day 1**: The child started having diarrhoea and vomiting. The mother took her child to a nearby public dispensary. At the dispensary, mother was given metronidazole tablets to give the child at home.

**Day 2**: Observing no improvement in the child, the next morning mother went back to the same dispensary. This time she was given one packet of oral rehydration solution and was told to continue giving metronidazole tablets as well.

**Day 3**: The diarrhoea and vomiting was getting worse. The child was getting weaker and started breathing fast. The mother decided to go straight to the health centre (next higher level facility). On arrival, she was ordered by the clinician to go and buy an injection (antibiotic) from the local drug store which she was told was going to help the fast breathing. After the child had received the injection she was then asked to take her to be investigated for malaria at a nearby private laboratory. There she paid, had the child tested and brought back the results which revealed malaria parasites. She was then told to go and buy quinine syrup. Later on the same day, the mother was told that her child's condition wasn't improving and that she should go to buy a second drug (antibiotic).

**Day 4**: The child was getting worse and was breathing very badly. The mother was sent to buy a third drug (antibiotic). The mother requested to be referred to the district hospital, but she was told even there they would give the child the same drug so she should rather go and buy the drug as advised. Later in the evening the quinine syrup the mother was giving her child by mouth was coming back through the nose. By that time the child could no longer breastfeed or eat but was still conscious. That is when she was referred the child to the district hospital. The mother was given a driver to bring her and her child to the district hospital, but no nurse was willing to accompany her. The child died 8 hrs after admission

Similar experiences of inadequate care were shared by other informants. In the following section the different deficiencies related to investigations, drugs, qualified health staff and services are further illustrated through the health seeking accounts of care takers.

### Lack of diagnostic facilities

In line with the quantitative results, unavailability of investigations emerged as the main reason for bypassing PHC facilities among caretakers interviewed qualitatively. Most caretakers were keen to get their children investigated so that they could be sure of which disease their children were suffering from before receiving treatment. The following quote illustrates:

"... *even if you take your child there, there is no service. If you arrive with a child with fever she will just be given ALU (current first line anti - malaria drug). There is no investigation that can tell you what's wrong with your child." *(Bypasser, Kwamhosi village, Muheza district).

In this malaria endemic area, the most important investigations as perceived by the interviewees was the tests for malaria and haemoglobin level. At PHC facilities in Tanzania these investigations are almost always unavailable [19]. The expectation of no investigations for malaria was reported to be a major reason for bypassing such facilities as expressed by the caretaker in the quote below:

*"We usually go there, and it's free but sometimes they don't have malaria investigation. They tell you to go and do it at a private hospital and come back with results. I find it bothersome so I decided to go straight to the private hospital..." *(Bypasser, Majengo village, Korogwe district).

As a way of dealing with lack of diagnostic facilities at the PHC facilities, one caretaker reported to have his child investigated elsewhere before going to the public dispensary:

*"I started at a research centre because I wanted to know what was wrong with my child and whether he had malaria, there they have investigations. After they discovered that he had malaria that's when I took him to the public dispensary to get treatment..." *(Non-bypasser, Kerenge village, Korogwe district)

### Lack of drugs

The problem of lack of drugs at PHC facilities is well illustrated as one of the 'lacks' in the mother's account presented in the case above. In addition, other caretakers who utilized their nearer PHC facilities reported:

"... *After arriving at the dispensary we were told to go buy intravenous fluids, because they had none. They gave the baby one drip, the other one wasn't finished and that's when they told us to go to the district hospital*" (Non-bypasser, Makiyumbi village, Muheza district)

Even a simple drug like paracetamol was sometimes not provided as reported by one caretaker who requested it at a health centre:

"*Back there, they didn't give me any help, even paracetamol. Because my child had fever, we requested for paracetamol but they told us to go and buy. But we decided to go straight to look for transport to take us to this hospital as we were afraid we might be late and lose the child, it was around 8.30 pm..." *(Non-bypasser, Kwadoya Village, Korogwe district).

The above caretaker presented at the district hospital with a convulsing, unconscious child. The child was diagnosed with severe malaria and received emergency care including blood transfusion and survived.

### Lack of qualified health workers

Unavailability of qualified health workers at PHC facilities was mentioned by some caretakers as a reason for their decision to bypass such facilities. This was encountered in two situations, either no health worker was available or that the trusted health worker was no longer available at a given facility:

*"I didn't take him there because there is no one to give drugs. The woman (nurse) has gone for a seminar and the man (clinical officer) is away, I guess he has gone to Tanga. I was told drugs are there but nobody to give you"*. (Bypasser; Kwamndolwa village - Korogwe district).

Another caretaker from the other district had these to say:

*"You know at the dispensary, those times Dr. N was around. At least when you went he would examine the child, I think he also has left. Now when you go there someone just looks at you and prescribes drugs for you, they tell you they have no investigations. So I decided to come straight here where I can get investigations." *(Bypasser, Kwafungo village, Muheza district).

Lack of qualified health workers at PHC facilities was as also reported to be encountered by a care-taker who utilized her nearer facility:

*"The main doctor is no longer there, he was brought to this hospital. Back there, we have remained with no one. I was given this drug to give at home but after seeing the way my child was breathing I decided not to give but come straight here" *(non-bypasser from Kerenge village - Korogwe district)

### Lack of services on weekends and holidays

Most dispensaries and some health centres in Tanzania do not operate during weekends and caretakers are forced to turn to other available options of care when need arises. As a result children may not receive appropriate care, as the case below illustrates.

#### Case 2

A mother was seen at the district hospital 3 days after the onset of symptoms of her 46 months old female child. On admission the child was unconscious and was diagnosed with severe malaria. Below is the mother's account of what happened.

**Day 1**: Mother was worried that her child was weak and did not eat well. At night the child developed fever.

**Day 2**: Next day the mother took the child to a private dispensary. The clinician diagnosed malaria and treated the child with SP (old first line anti-malarial drug, no longer in use).

"Usually I don't take my child there, that day I went because it was Saturday and the public hospital wasn't operating", the mother reported.

The same night the child developed high fever and started making noise with abnormal movements (fits). The mother thought it was the result of the drug fighting the malaria parasites and gave her child paracetamol. The fever went down and the child continued to sleep.

**Day 3**: The next morning the child seemed OK. She was playing but did not take much of her tea and chapati for breakfast. In the afternoon the child refused to eat anything.

" We prepared ugali for lunch and when she refused I cooked her rice which she likes but still she did not eat even a little, then I told myself my child must be real ill", the mother reported. Later during the day, the child's condition became worse as reported below by the mother; "Around 2 pm, she started making noise, very much noise, and was shaking strongly (convulsions). I tried to make her stand but she couldn't, I tried to make her sit, she couldn't either. In a short while her face changed completely and was not that of Mahija (child's name). I was so afraid and I asked myself, "What's wrong with my child?" I told her father who told me to get prepared to go to the hospital and that's when we came here..."

The child died later the same day.

## Discussion

The study found that almost 6 in 10 caretakers seen at the district hospital had bypassed their nearer PHC facilities. The majority had opted to give drugs purchased from local and drug shops prior to coming to the district hospital, a practise that poses several dangers and may often lead to delays in seeking appropriate care. First of all, the drugs are given without prior examination of the child which may result in ineffective treatment. Secondly, the inexperienced prescribers at local and drug shops may give wrong dosage, which may lead to ineffective or harmful effects of the drugs given. Furthermore, most of these shops are very poorly regulated and sometimes sell unregistered and expired drugs [20].

The main reason given for bypassing the PHC facilities in our study was the lack of diagnostic facilities at such facilities, particularly lack of equipments to test for malaria and blood haemoglobin level. The above two investigations are commonly unavailable at most PHC facilities in Tanzania [19]. Health workers generally make a diagnosis based on symptoms only and tend to prescribe anti-malarial drugs to most children with a history of fever. Malaria prevalence has decreased during the last decade in Tanzania as well as in other parts of Sub-Saharan Africa [21]. This may indicate that if not properly investigated, many children with fever may wrongly be diagnosed as malaria cases, and the treatment given may then not lead to recovery. Care-takers with such experiences may then tend to bypass these facilities the next time they seek care.

The second most common reason given for bypassing PHC facilities was the lack of drugs. Having travelled for some distance to reach a dispensary or a health centre, some of which charge unofficial fees for consultation [22], caretakers reported that their children commonly had not undergone any investigation. Furthermore, they were often told that drugs were out of stock and therefore given prescriptions to buy them elsewhere. In the first case described above, the caretaker was told to buy drugs three times, which in addition to taking her precious time, was very costly. The mother spent a lot of money and ended up loosing her child. This could explain why many caretakers in our study opted to use other options of care and bring children straight to the hospital if the condition did not improve. It may as well explain the ongoing reported preference for the "over-the counter drugs" as the first option of care reported from several previous studies [23-26].

Another reason for bypassing PHC facilities given by caretakers in our study was lack of qualified personnel at such facilities, which is not a new finding. The main problem has been poor distribution where a majority of health staff are found in towns and big cities leaving the rural areas under-staffed [27]. A recent survey conducted in six districts of Northern Tanzania concluded that there were actually adequate human resources for health care provision in the area according to national standards. However, most qualified staff were concentrated in a few centralized locations [28].

Preference for higher level facilities has been reported in several other studies in Tanzania [11,12], as well as from other low-income countries [7-9]. In a study conducted in Tanzania, 44% of the women seeking birth care had bypassed their nearest facility while 59.8% of women who lived in a village with a functioning health facility had delivered at home [11]. In this study, the women reported that quality of care (e.g. best provider, availability of drugs) and a greater trust in health workers at the facility chosen for delivery were the main reasons for selecting the facility. Results from a household survey carried out in Lushoto district in Tanzania showed that patients bypassed their lower level of health care to seek hospital treatment because of poor quality of services and poor availability of drugs [12].

Results from a population based discrete choice experiment to evaluate factors influencing women's preferences for place of delivery in rural Tanzania supports the above findings (29). The experiment established that the most important facility attributes influencing women's preferences for place of delivery were a respectful provider attitude and availability of drugs and medical equipment. Policy simulations suggested that if these attributes were improved at existing facilities, the proportion of women preferring facility delivery would rise from 43% to 88% [29].

Poor quality of services at PHC facilities has previously been reported from several other studies in Tanzania [12,30-33]. In one of the studies, it was found that a typical dispensary had a 40% probability of having the required level of drugs for injections and shortages of diagnostic and dressing equipments were very common [31]. In the same study above it was found that, the only real curative care advantage of health centres compared to dispensaries was their laboratory services. However, these laboratories were characterised by frequent shortage of reagents and other materials. Drugs availability in health centres was found to be even worse as compared to dispensaries. In a study carried out in Morogoro rural and Kilombero region, health centres and dispensaries were characterized by very long waiting times and shortage of staff and medicines [33].

From Kenya, Audo et al. reported that between 46.3% and 59.5% of mothers in a rural district had bypassed their lowest level hospitals in favour of district or provincial hospitals when seeking antenatal care, child immunizations and other child health services [7]. The most frequent reasons for bypassing PHC facilities given by the women in this study were poor care (21%), lack of drugs and supplies (17%) and poor laboratory services (12%). In Sri-Lanka, 66.5% of survey respondents with a minor or major illness in the past month reported bypassing their nearest health facility [8].

Our quantitative survey found shorter travel time, lower disease severity and shorter duration of symptoms to be associated with increasing frequency of bypassing a PHC facility for child care (Table 2). Several previous studies have shown that distance is an important barrier to health care access [34,35]. It is worth noting that in our study, 52.4% of caretakers who reported to have travelled for two or more hours to reach the district hospital had bypassed their nearer PHC facilities. The substantial higher proportion of children with severe disease among non-bypassers (83.3%) as compared to bypassers (48.7%) could in part be a result of selection bias. Children with non-severe disease and who improved after utilizing their nearer PHC facilities obviously needed not to go to the district hospital and are hence under-represented. However, the higher disease severity and longer delays among those who first visit PHC facilities may indicate that treatment given at these facilities is not appropriate and that the use of these facilities may delay caretakers from seeking appropriate care. The findings from the qualitative interviews support this explanation.

The observation that the majority of children (81.7%) seen with non-severe disease at the district hospital had actually bypassed their nearer PHC facilities, suggests strong over-utilization of higher level facilities that are intended to treat severe cases referred from PHC facilities. When PHC facilities are bypassed, children are treated at higher costs than necessary, PHC facilities become under-utilized and higher level facilities become overburdened and diverted from their primary purpose of providing specialized care for more complicated cases [36,37].

### Methodological strengths and limitations

This study inquired care-seeking information related to the current child illness. By using this approach, we believe we were able to pick up easily forgettable care-seeking information which community based studies may miss, often relying on past illness episodes of preceding weeks. The use of both quantitative and qualitative research methods which supported one another strengthens the validity of our findings.

The study was hospital-based and thus prone to selection bias. Caretakers who utilize PHC facilities or other options of care and did not proceed to the district hospitals could not be studied. Therefore, only the frequency of bypass among those reaching the district hospitals could be established, and not the overall frequency of bypass based on all who went for treatment at any facility in the area. However the bypassing frequency established in our study was somewhat similar to the bypassing frequencies reported in community-based studies referred to above. In addition, this was the most practical way to study bypass in relation to severe disease, since disease severity could possibly not be well established from merely a history of previous sickness episode at household level.

Owing to the ethical consideration that the interviews were only to be conducted after the child had received emergency care and was stable, very severely ill children who died within a few hours after admission were missed. However we believe this did not affect our findings as the numbers were small (we missed only 24 children who were confirmed to have died from malaria, pneumonia or diarrhoea).

We had no refusals, neither for the quantitative nor the qualitative study. This may be partly because the study was hospital-based and interviews were done among caretakers of sick children by clinicians who were involved in the treatment of the children. Furthermore, care-takers may have had no objection to participate because the study involved no alteration in the standard care and only inquired care-seeking information which is usually obtained in any normal consultation.

## Conclusions

Our study has shown that people are willing to travel long distances to reach facilities perceived to provide services of better quality. This is a major equity issue indicating that quality health care is less accessible to the economically disadvantaged population who can not afford travelling costs to access quality care at higher level hospitals. Improving the quality of care at PHC facilities could also reduce delays in seeking appropriate care. Making simple investigations like rapid malaria diagnostic tests available and securing essential drugs at PHC facilities would improve case diagnosis and management which in turn may increase trust in the care provided at these facilities. Hence, we argue that in a situation of scarce resources, strengthening already existing PHC facilities to provide better quality services to rural populations should be prioritised before increasing the number of such facilities. We however acknowledge that provision of drugs and diagnostic facilities alone may not necessarily result in increase in services uptake, other factors such as provider-user interrelations also need to be addressed.

## Abbreviations used

PHC: Primary Health Care; OPD: Out-Patient Department; WHO: World Health Organization; ORS: Oral Rehydration Solution; ALU: Artemether Lumefantrine (first-line antimalaria drug).

## Competing interests

The authors declare that they have no competing interests.

## Authors' contributions

CK planned and wrote the protocol, collected and analysed data, and was the lead author of the manuscript. GK had substantial contribution to the protocol development as well as data analysis and writing the manuscript. SGH and KMM supervised data analysis and contributed in writing the manuscript. All authors have read and approved the final version.

## Pre-publication history

The pre-publication history for this paper can be accessed here:

http://www.biomedcentral.com/1472-6963/11/315/prepub
